# *In Vitro* Inhibition of Human Hepatic and cDNA-Expressed Sulfotransferase Activity with 3-Hydroxybenzo[*a*]pyrene by Polychlorobiphenylols

**DOI:** 10.1289/ehp.7837

**Published:** 2005-02-24

**Authors:** Li-Quan Wang, Hans-Joachim Lehmler, Larry W. Robertson, Charles N. Falany, Margaret O. James

**Affiliations:** ^1^Department of Medicinal Chemistry, University of Florida, Gainesville, Florida, USA;; ^2^Department of Occupational and Environmental Health, College of Public Health, University of Iowa, Iowa City, Iowa, USA;; ^3^Department of Pharmacology and Toxicology, University of Alabama, Birmingham, Alabama, USA

**Keywords:** 3-hydroxy-benzo[*a*]pyrene, human liver cytosol, inhibition of sulfonation, polychlorobiphenylols, SULT1A1*1, SULT1A1*2, SULT1E1

## Abstract

Sulfonation is a major phase II biotransformation reaction. In this study, we found that several polychlorobiphenylols (OH-PCBs) inhibited the sulfonation of 3-hydroxybenzo[*a*]pyrene (3-OH-BaP) by human liver cytosol and some cDNA-expressed sulfotransferases. At concentrations > 0.15 μM, 3-OH-BaP inhibited its own sulfonation in cytosol fractions that were genotyped for *SULT1A1* variants, as well as with expressed SULT1A1*1, SULT1A1*2, and SULT1E1, but not with SULT1A3 or SULT1B1. The inhibition fit a two-substrate kinetic model. We examined the effects of OH-PCBs on the sulfonation of 0.1 or 1.0 μM 3-OH-BaP, noninhibitory and inhibitory substrate concentrations, respectively. At the lower 3-OH-BaP concentration, OH-PCBs with a 3-chloro-4-hydroxy substitution pattern were more potent inhibitors of cytosolic sulfotransferase activity [with concentrations that produced 50% inhibition (IC_50_) between 0.33 and 1.1 μM] than were OH-PCBs with a 3,5-dichloro-4-hydroxy substitution pattern, which had IC_50_ values from 1.3 to 6.7 μM. We found similar results with expressed SULT1A1*1 and SULT1A1*2. The OH-PCBs were considerably less potent inhibitors when assay tubes contained 1.0 μM 3-OH-BaP. The inhibition mechanism was noncompetitive, and our results suggested that the OH-PCBs competed with 3-OH-BaP at an inhibitory site on the enzyme. The OH-PCBs tested inhibited sulfonation of 3-OH-BaP by SULT1E1, but the order of inhibitory potency was different than for SULT1A1. SULT1E1 inhibitory potency correlated with the dihedral angle of the OH-PCBs. The OH-PCBs tested were generally poor inhibitors of SULT1A3- and SULT1B1-dependent activity with 3-OH-BaP. These findings demonstrate an interaction between potentially toxic hydroxylated metabolites of PCBs and polycyclic aromatic hydrocarbons, which could result in reduced clearance by sulfonation.

Polycyclic aromatic hydrocarbons (PAHs) and polychlorinated biphenyls (PCBs) are two classes of environmentally prevalent pollutants. PAHs are formed through the combustion of fossil fuels and the burning of organic materials (Dipple 1985). PCBs were first produced industrially in the middle of the last century for their desirable dielectric properties (Erickson 2001) and remain in the environment because of their continued use, because of their release from waste sites, and because many congeners are slowly degraded. The more lipophilic PAHs and PCBs are often found in the same environmental samples, such as soils and sediments, and are bio-transformed in animals by similar pathways (James 2001).

Of the PAHs, benzo[*a*]pyrene (BaP) is a well-studied chemical carcinogen, which is metabolized by cytochrome P-450 (CYP) to a variety of products (Dipple 1985). These include 3-hydroxybenzo[*a*]pyrene (3-OH-BaP), a major metabolite of BaP in humans and animals, which has estrogenic properties and binds to hemoglobin (Charles et al. 2000; Sugihara and James 2003). Hydroxylated PAH metabolites such as 3-OH-BaP are substrates for glucuronidation and sulfonation, catalyzed by one or more of the UDP-glucuronosyltransferases and 3′-phosphoadenosine 5′-phosphosulfate (PAPS)-dependent sulfotransferases (SULTs), respectively (James et al. 2001). Sulfonation is considered a detoxification pathway for 3-OH-BaP.

PCBs have several metabolites of toxicologic importance, including the polychlorobiphenylols (OH-PCBs), which are formed *in vivo* from CYP-dependent mono-oxygenation of PCBs (James 2001). Although they are slightly more hydrophilic than are the parent PCBs, several OH-PCBs are eliminated slowly (Klasson-Wehler et al. 1993). People who are highly exposed to PCBs through the diet typically have OH-PCBs in their blood, some bound to plasma proteins (Guvenius et al. 2003; Sandau et al. 2000). Several OH-PCB congeners interact with components of the endocrine system, potentially interfering with thyroid hormone and estrogen function (Lans et al. 1993; Safe 1994; Schuur et al. 1998). Although the OH-PCBs have low affinities for both α and β estrogen receptors, some OH-PCBs are strikingly potent inhibitors of human estrogen sulfotransferase (SULT1E1), with sub-nanomolar concentrations that produced 50% inhibition (IC_50_) (Kester et al. 2000). This suggests that OH-PCBs may be indirectly estrogenic by increasing estradiol bioavailability in target tissues. As well as possibly causing toxicity by inhibiting the sulfonation of hormones, several OH-PCBs inhibited the sulfonation and glucuronidation of the PAH metabolite 3-OH-BaP in channel catfish intestine (van den Hurk et al. 2002).

Sulfonation is an important phase II conjugation pathway for the detoxification of xenobiotics as well as the modulation of endogenous compounds such as thyroid hormones, steroids, and neurotransmitters (Coughtrie et al. 1998). One or more members of a superfamily of cytosolic SULT enzymes catalyze these reactions (Blanchard et al. 2004). SULT1A1, SULT1B1, and SULT1E1 are the major phenol sulfotransferases expressed in human liver, with SULT1A1 (also known as ST1A3) found at the highest concentration (Honma et al. 2002). SULT1A3 is expressed in the gut but is present in very low concentrations in adult human liver (Richard et al. 2001). Genetic polymorphisms are known for *SULT1A*: a G^638^→A transition leading to an Arg^213^→His exchange in the protein was observed with a frequency of 33.2% in Caucasian subjects, 8% in Chinese, and 29.4% in African Americans (Carlini et al. 2001). SULT1A1*His (SULT1A1*2) was a less thermostable protein than SULT1A1*Arg (SULT1A1*1), and some authors have reported that the SULT1A1*2 variant is less catalytically active (Ozawa et al. 1998; Raftogianis et al. 1997).

Because people are frequently coexposed to PAHs and PCBs, we wished to determine if OH-PCBs would inhibit 3-OH-BaP sulfonation in human liver (HL) cytosol and, if so, whether the inhibition was isozyme selective. We used cDNA-expressed human SULT1A1*1, -1A1*2, -1A3, -1B1, and 1E1 isozymes, which we expected would use 3-OH-BaP as substrate. We genotyped the HL cytosol fractions used in this study, with respect to the common SULT1A1 polymorphic variants, to examine the possibility that OH-PCBs would affect their activity differently. These studies were conducted with a series of predominantly *para*-OH-PCBs.

## Materials and Methods

### Materials.

**Figure 1:**
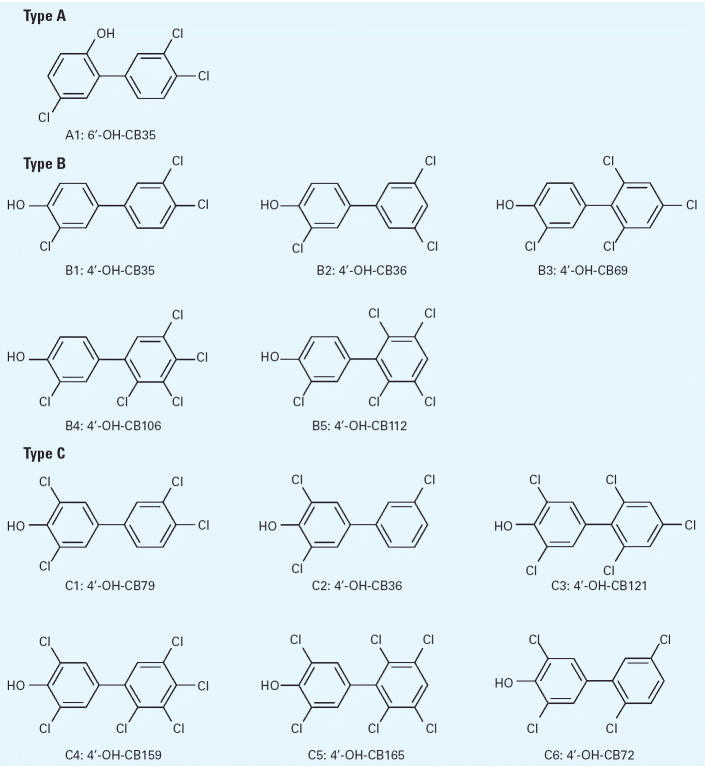
Structures of the hydroxylated PCBs used in this study. Type A, hydroxy without a flanking chlorine atom; type B, *para*-hydroxy with one flanking chlorine atom; type C, *para*-hydroxy with two flanking chlorine atoms.

The structures of the OH-PCBs used in this study are shown in Figure 1. In naming these OH-PCBs, we followed the recommendation of Maervoet et al. (2004) to name them as metabolites of PCBs, referring back to the Ballschmiter and Zell numbering system for PCBs (Ballschmiter and Zell 1980). The 6′-OH-CB35 (A1), 4′-OH-CB35 (B1), 4′-OH-CB36 (B2), 4′-OH-CB79 (C1), and 4-OH-CB36 (C2) were synthesized by Suzuki coupling as described previously (Bauer et al. 1995; Lehmler and Robertson 2001). We verified the structures of each of these OH-PCBs by ^1^H and ^13^C-nuclear magnetic resonance spectroscopy, Fourier transform infrared spectroscopy, and gas chromatography–mass spectrometry (GC-MS). We found that each OH-PCB was > 99% pure by GC-MS analysis (Mass Spectrometry Facility, University of Kentucky, Lexington, KY), combustion analysis (Atlantic Microlab, Atlanta, GA), and thin-layer chromatography. The 4′-OH-CB69 (B3), 4′-OH-CB106 (B4), 4′-OH-CB112 (B5), 4′-OH-CB121 (C3), 4′-OH-CB159 (C4), 4′-OH-CB165 (C5), and 4′-OH-CB72 (C6) were purchased from AccuStandard (New Haven, CT). S.S. Singer (University of Dayton, Dayton, OH) supplied the PAPS. We purchased ^35^S-PAPS, 3.05 μCi/nmol (99.1% pure), from PerkinElmer Life Science (Boston, MA). Benzo[*a*]pyrene-3-sulfate (BaP-3-SO_4_) and 3-hydroxybenzo[*a*]pyrene (3-OH-BaP) were purchased from the NCI Chemical Carcinogen Reference Standard Repository (Midwest Research Institute, Kansas City, MO). We obtained *Hae*II from Fisher Scientific (Atlanta, GA) and *Taq* DNA polym-erase, along with other polymerase chain reaction (PCR) reagents, from Promega (Madison, WI). Integrated DNA Technologies (Coralville, IA) supplied primers for use in genotyping. We purchased the highest available grade of other reagents from Fisher Scientific (Atlanta, GA) and Sigma Chemical Company (St. Louis, MO).

### Physicochemical properties of the OH-PCBs.

We calculated the structural characteristics of dihedral angle, molecular volume, molecular surface area, p*K*_a_, log *P*, and log *D* at pH 7.0 with MM2* using GB/SA water solvent continuum as implemented by MacroModel 5.0 (Schrödinger, Portland, OR) and described previously by Tampal et al. (2002).

### Cytosolic preparations.

F.P. Guengerich (Vanderbilt University) kindly donated the samples of human liver, which were procured from organ donors (Guengerich 1995). We prepared liver cytosolic fractions from four livers by standard methods and stored aliquots at −80°C until use (Wang et al. 2004). We used three or four of these cytosol fractions in each experiment.

### SULT1A1 *genotype determination.*

We used a genomic DNA isolation kit (EASY-DNA; InVitrogen, Carlsbad, CA) to extract genomic DNA from samples of the individual human livers used in this study. We used a published method to detect the *SULT1A1* polymorphism status of each liver (Nowell et al. 2000; Ozawa et al. 1998). Amplified DNA fragments were digested with *Hae*II, and the fragments were resolved on 2% (weight/volume) agarose gels. Fragments from individuals homozygous for SULT1A1*1 exhibited two bands, visualized by ultraviolet transillumination, whereas DNA fragments from individuals homozygous for SULT1A1*2 were not cleaved by this enzyme and exhibited one band.

### Expression and partial purification of SULT isozymes.

The expression of human SULT1A1*1, SULT1A3, SULT1B1, and SULT1E1 in *Escherichia coli* has been described previously (Dajani et al. 1998; Wang et al. 1998). We grew *E. coli* cells containing the respective sulfotransferase genes as described previously (Falany et al. 1990, 1994), and prepared 100,000*g* supernatant fractions for use in inhibition studies and for partial purification of the SULT enzymes. We purchased expressed SULT1A1*2 cytosolic extract from PanVera (Madison, WI) and used it as supplied.

The 100,000*g* supernatant fractions of SULT1A1*1, SULT1A3, SULT1B1, and SULT1E1 were partially purified by chromatographic methods (Falany et al. 1990, 1994). After the last step, a 3′-phosphoadenosine 5′-phosphate (PAP)-agarose affinity column, we dialyzed the fractions eluted with PAP with three changes of buffer to remove PAP before the assay of SULT activity with 3-OH-BaP as substrate. We analyzed active fractions by SDS-PAGE (Laemmli 1970) to assess the purity of each SULT enzyme. We stained the gels with Coomassie R-250 reagent and determined the percentage of protein present as each respective SULT enzyme by scanning densitometry.

### Kinetic analysis of 3-OH-BaP sulfonation.

We determined SULT activity with 3-OH-BaP as substrate by a fluorimetric assay of BaP-3-SO_4_ product formation, as described previously (Wang et al. 2004). We ensured that the formation of BaP-3-SO_4_ was linear for time and protein and did not exceed 10% of the added 3-OH-BaP with each of the enzyme sources used. Duplicate tubes were prepared for each incubation condition. We examined the kinetics of sulfonation in three liver cytosol fractions by systematically varying the concentration of 3-OH-BaP or PAPS. When the variable substrate was 3-OH-BaP, we used 12 concentrations in the range from 0.035 to 2.00 μM, and the concentration of PAPS was kept constant at 10 μM. When we varied PAPS, we used 7 concentrations from 0.157 to 10.0 μM and kept the concentration of 3-OH-BaP constant at 0.100 μM.

We determined the kinetic parameters for 3-OH-BaP sulfonation by partially purified preparations of the cDNA-expressed SULT isozymes under incubation conditions similar to those used for liver cytosol. For SULT1A1*1 and -1A1*2, we used seven substrate concentrations in the range from 5 to 100 nM; for SULT1E1 we used six 3-OH-BaP concentrations from 15.6 to 1,000 nM; and for SULT1A3 and 1B1 we used seven concentrations of 3-OH-BaP from 0.25 to 5.0 μM.

### Inhibition of SULT activity by OH-PCBs.

To assess inhibition of 3-OH-BaP SULT activity, we prepared stock solutions of OH-PCBs in dimethyl sulfoxide (DMSO) and added aliquots to incubation mixtures such that the final concentration of OH-PCB was in the range of 0.01–200 μM and the DMSO concentration did not exceed 0.5% (vol/vol). For each OH-PCB, we examined the concentration dependence of inhibition with three liver cytosol fractions, as well as with cytosol fractions from the *E. coli* expressing SULT1A1*1, SULT1A3, SULT1B1, and SULT1E1, and the purchased Sf-9 cytosol fraction (PanVera, Madison, WI) containing SULT1A1*2. For studies with HL cytosol, SULT1A1*1, and SULT1E1, we examined two concentrations of 3-OH-BaP, 0.1 μM and 1.0 μM. For studies with SULT1A1*2, we examined only 0.1 μM 3-OH-BaP, a concentration that did not elicit substrate inhibition. For studies with SULT1B1, we used only 1.0 μM 3-OH-BaP because this enzyme had very low activity at 0.1 μM 3-OH-BaP and did not exhibit substrate inhibition. Examination of the effect of 50 μM concentrations of several OH-PCBs on the activity of SULT1A3, measured with 1.0 μM 3-OH-BaP, revealed little inhibition, so no further concentrations were studied.

### Kinetics of inhibition.

To study the type of inhibition produced by OH-PCBs, we used 4′-OH-CB112 (B5) as a model inhibitor. We prepared four sets of assay tubes containing HL cytosol and varying amounts of 3-OH-BaP from 35 to 150 nM: one set (control) contained no 4′-OH-CB112; the other sets contained 0.25 μM, 0.5 μM, or 1.0 μM 4′-OH-CB112.

### Data analysis.

We calculated the enzyme kinetic parameters from studies with variable concentrations of 3-OH-BaP using nonlinear regression analysis and GraphPad 4.0 software (GraphPad Software, San Diego, CA). We selected the built-in Michaelis-Menten equation for most analyses. Where we found evidence of 3-OH-BaP substrate inhibition, we fit the data into an equation derived from a two-substrate model (Zhang et al. 1998):


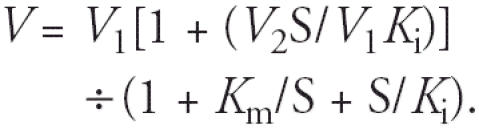


This equation denoted the constant for binding of the first substrate (S) molecule as *K*_m_ and the second substrate molecule as *K*_i_. *V*_1_ is the maximum rate for the noninhibitory substrate concentration range, and *V*_2_ is the minimum rate in the inhibitory substrate concentration range

We calculated the effects of OH-PCBs on 3-OH-BaP SULT activity as percentage inhibition compared with the controls without an inhibitor. We obtained IC_50_ values by fitting log OH-PCB concentration and percent control activity to a sigmoidal curve. We examined the relationships between IC_50_ and physicochemical properties of the OH-PCBs by linear correlation analysis. We calculated the inhibitory constant (*K*_i_) from the kinetic studies with 4′-OH-CB112 by means of Dixon plots and plots of *K*_m_/*V*_max_ against inhibitor concentration (Cornish-Bowden 1995).

## Results

### SULT1A1 *genotype of the liver donors.*

**Figure 2:**
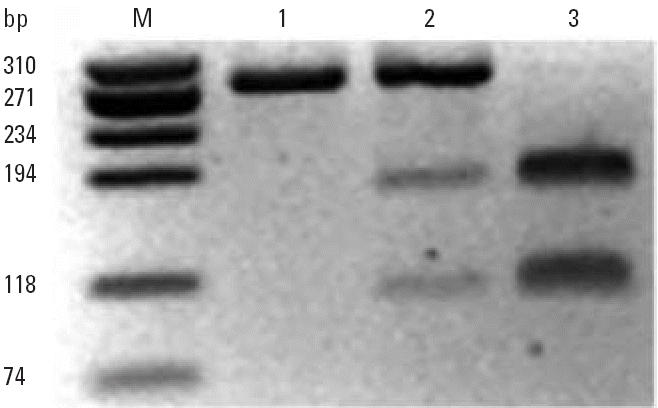
Detection of SULT1A1*1/*2 alleles by restriction fragment length polymorphism analysis. Lane M, marker; lane 1, SULT1A1*2/*2 homozygous; lane 2, SULT1A1*1/*2 heterozygous; lane 3, SULT1A1*1/*1 homozygous. Specific PCR products were generated and digested with *Hae*II as described in “Materials and Methods.”

We found that the HL cytosols used were from individuals with different *SULT1A1* genotypes, as determined by PCR amplification of the region of the *SULT1A* gene flanking the polymorphic base pair. The G to A mutation in *SULT1A1* removed the restriction site for the endonuclease *Hae*II. As shown in Figure 2, an individual homozygous for the SULT1A1*2 allele did not have the *Hae*II restriction site, and the PCR product was not cleaved (lane 1). The PCR product from the individual homozygous for SULT1A1*1 showed complete cleavage by *Hae*II, generating two fragments of approximately 100 and 181 bp (lane 3). Enzymatic digestion of the PCR product from the heterozygote (SULT1A1*1/*2) generated one band of 281 bp and the two fragments of 100 and 181 bp (lane 2). Thus, the individual liver designated HL 1 was homozygous for the SULT1A1*1 allele, HL 2 was heterozygous, and HL 3 was homozygous for the SULT1A1*2 allele.

### Sulfonation of 3-OH-BaP by HL cytosol and expressed human SULT isoforms.

**Figure 3:**
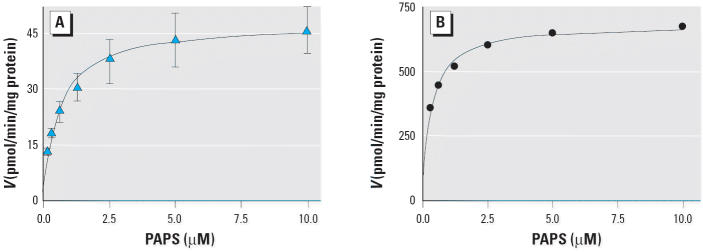
Rates of sulfonation of 3-OH-BaP (0.1 μM) in the presence of varying concentration of PAPS (0.125–10 μM) in HL cytosol (*A*) and cytosol of SULT1A1*2 (*B*). Data in (*A*) are given as the mean ± SD of three experiments.

Initial studies of the sulfonation of 3-OH-BaP by HL cytosol revealed that concentrations of 3-OH-BaP > 0.15 μM resulted in a decrease in activity. To find a saturating concentration of PAPS, we conducted incubations in the presence of 0.1 μM 3-OH-BaP and varying concentrations of PAPS. The data fit the Michaelis-Menten equation, with an apparent *K*_m_ of 0.56 ± 0.09 μM and a *V*_max_ of 48 ± 2 pmol/min/mg protein (mean ± SD; *n* = 3). The dependence of activity upon PAPS concentration in expressed human SULT1A1*2, in the presence of 0.1 μM 3-OH-BaP, also followed Michaelis-Menten kinetics. The apparent *K*_m_ was 0.32 μM, and *V*_max_ was 684 pmol/min/mg protein. As shown in Figure 3, cytosol and the expressed enzyme were saturated by a PAPS concentration of 10 μM, and we used this concentration in subsequent studies.

**Figure 4:**
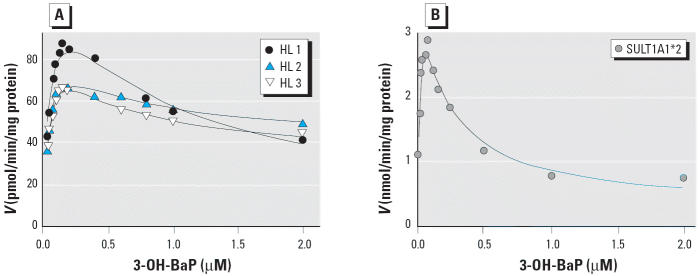
Partial substrate inhibition by 3-OH-BaP in HL cytosol from three individuals (*A*) and SULT1A1*2 (*B*).

We conducted detailed studies of the effect of a range of 3-OH-BaP concentrations up to 2 μM on reaction rates with HL cytosol and expressed human SULT1A1*2. We obtained preliminary estimates of the kinetic constants *K*_m_ and *V*_1_ by fitting the initial rates of sulfonation at concentrations < 0.15 μM 3-OH-BaP to the Michaelis-Menten equation. We then obtained the values of *K*_i_ and *V*_2_ through constraining *K*_m_ using the equation of Zhang et al. (1998). We also analyzed data by constraining *V*_1_, but a better fit was found when constraining *K*_m_. Figure 4A shows how the data fit this equation for three individual HL cytosols. Kinetic studies with expressed SULT1A1*2 revealed substrate inhibition with the single enzyme (Figure 4B). Table 1 shows values for *K*_m_, *K*_i_, *V*_1_, and *V*_2_ for each HL cytosol and the expressed SULT1A1*2. The expressed enzyme showed a lower value for *K*_m_ (0.022 μM) and *K*_i_ (0.16 μM) than did any of the HL cytosols.

Table 2 shows the results of kinetic studies with the other expressed human enzymes. The values shown in Table 2 are from substrate concentration ranges in which the data fit the Michaelis-Menten equation. SULT1A1*1 and SULT1E1 showed substrate inhibition at concentrations of 3-OH-BaP > 0.15 μM, but detailed kinetic analyses at inhibitory concentrations was not conducted with these expressed enzymes. We found that SULT1A1*1 had an apparent *K*_m_ (0.018 μM) similar to that found with SULT1A1*2 (0.022 μM). SULT1E1 also had high affinity for 3-OH-BaP, with an apparent *K*_m_ of 0.05 μM. SULT1A3 and SULT1B1 did not exhibit substrate inhibition over a concentration range up to 5 μM and showed much higher apparent *K*_m_ values for 3-OH-BaP. These expressed enzyme preparations were partially purified, and SDS-PAGE showed they contained different percentages of the respective SULT enzymes (Table 2). The values shown for *V*_max_ were corrected for the percentage of each respective SULT isoform in the partially purified enzyme preparation.

### Inhibition of 3-OH-BaP sulfonation by OH-PCBs with HL cytosol.

**Figure 5:**
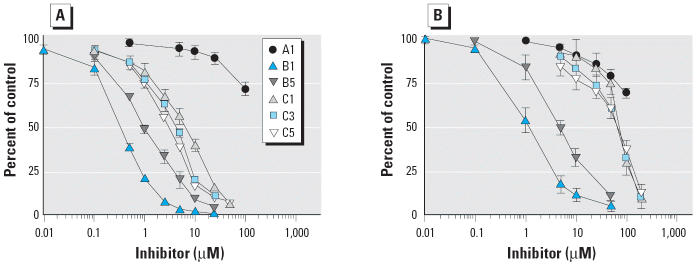
Inhibition of 3-OH-BaP sulfotransferase in HL cytosol by OH-PCBs. (*A*) 0.1 μM 3-OH-BaP. (*B*) 1.0 μM 3-OH-BaP. 3-OH-BaP sulfotransferase activity is given as percentage of control. Data given are the mean ± SD of three experiments. Structures of the tested OH-PCBs are shown in Figure 1.

The 4-OH-PCBs with one (B group) or two (C group) flanking chlorine substituents inhibited HL cytosolic 3-OH-BaP sulfotransferase activity in a concentration-dependent manner. Figure 5A shows inhibition curves from selected OH-PCBs in the presence of 0.1 μM 3-OH-BaP, and Figure 5B shows the same compounds studied with 1.0 μM 3-OH-BaP. Table 3 presents the IC_50_ values of 3-OH-BaP sulfotransferase activity with all the tested compounds, each at two concentrations of 3-OH-BaP. Compounds B1–B5 with the 3-chloro-4-hydroxy substitution pattern were potent inhibitors, with IC_50_ values ranging from 0.33 to 1.08 μM, when activity was measured with 0.1 μM 3-OH-BaP. The OH-PCBs with two chlorine atoms flanking the hydroxy group (C1–C6) were less potent inhibitors under these conditions (IC_50_, 1.31–6.71 μM; Table 3). The single 6-OH-PCB studied, A1, was a very weak inhibitor, with an IC_50_ of > 100 μM (Figure 5). When activity was measured with 1 μM 3-OH-BaP, a concentration at which substrate inhibition occurred, the measured IC_50_ values showed lower inhibitory potencies for all OH-PCBs, but especially so for the C group compounds, whose IC_50_ values ranged from 3 to 58.7 μM (Table 3).

### Inhibition of 3-OH-BaP sulfonation by OH-PCBs with cDNA-expressed SULTs.

**Figure 6:**
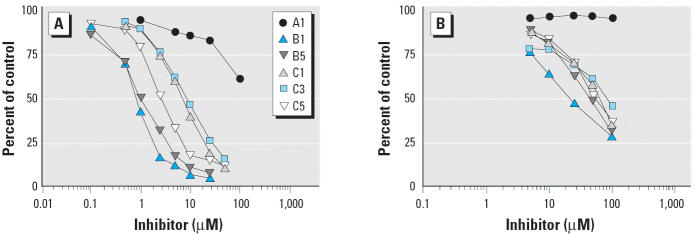
Inhibition of 3-OH-BaP sulfotransferase in SULT1A1*1 by OH-PCBs. (*A*) 0.1 μM 3-OH-BaP. (*B*) 1.0 μM 3-OH-BaP. 3-OH-BaP sulfotransferase activity is given as percentage of control. Data given are the mean ± SD of three experiments. Structures of the tested OH-PCBs tested are shown in Figure 1.

For SULT1A1*1, Figure 6A shows inhibition curves with selected OH-PCBs using 0.1 μM 3-OH-BaP, whereas Figure 6B shows results with a substrate concentration of 1.0 μM 3-OH-BaP. We found that 6′-OH-CB35 (A1) was a poor inhibitor of 3-OH-BaP sulfonation under both conditions of substrate concentration. When using 0.1 μM 3-OH-BaP, type B compounds (B1–B5) showed IC_50_ values ranging from 0.77 to 1.31 μM, whereas type C compounds (C1–C6) exhibited IC_50_ from 2.16 to 6.65 μM (Table 3). When using 1.0 μM 3-OH-BaP, the inhibitory potencies of the OH-PCBs were dramatically reduced. The IC_50_ values for type B OH-PCBs were reduced to 10.3–67.5 μM, and for type C OH-PCBs were 33.8 to > 100 μM (Table 3).

For SULT1A1*2, the IC_50_ of 6′-OH-CB35 (A1) was > 100 μM, as shown in Table 3. At 0.1 μM 3-OH-BaP, the IC_50_ ranged from 0.54 to 1.48 μM for type B (B1–B5) compounds and from 1.67 to 6.52 μM for type C compounds (C1–C6). When using 1.0 μM 3-OH-BaP, the OH-PCB IC_50_ was approximately 5 μM for type B (B1–B5) compounds and 50 μM for type C (C1–C6) compounds (data not shown).

**Figure 7:**
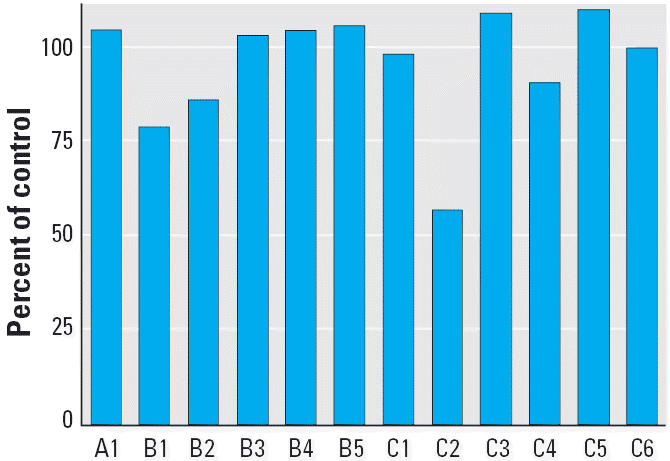
Inhibition of 3-OH-BaP sulfotransferase activity with SULT1A3 by OH-PCBs shown in Figure 1, each at 50 μM.

As shown in Figure 7, expressed SULT1A3 was not inhibited or was weakly inhibited by OH-PCBs when 3-OH-BaP was used at the noninhibitory concentration of 1.0 μM. Addition of 50 μM concentrations of compounds 6′-OH-CB35 (A1), 4′-OH-CB69 (B3), 4′-OH-CB106 (B4), 4′-OH-CB112 (B5), 4′-OH-CB121 (C3), 4′-OH-CB165 (C5), and 4′-OH-CB72 (C6) did not inhibit the sulfonation of 3-OH-BaP. Compounds 4′-OH-CB35 (B1), 4′-OH-CB36 (B2), 4′-OH-CB79 (C1), and 4′-OH-CB159 (C4) showed 2–20% inhibition at 50 μM, and 4-OH-CB36 (C2) produced 43% inhibition. Because SULT1A3 activity was poorly inhibited by 50 μM concentrations, we did not examine a range of concentrations of OH-PCBs.

Expressed SULT1B1 showed a quite different inhibitory interaction with OH-PCBs, compared with SULT1A1*1, SULT1A1*2, SULT1A3, and SULT1E1, in that 6′-OH-CB35 (A1) was a quite potent inhibitor (IC_50_, 4.72 μM) of 3-OH-BaP sulfonation (Table 3). Compounds B1 (4′-OH-CB35) and B4 (4′-OH-CB106) showed IC_50_ values of 16.76 and 17.45 μM, respectively. The other type B and type C OH-PCBs were weak inhibitors.

For SULT1E1, compound A1 (6′-OH-CB35) was a poor inhibitor of 3-OH-BaP sulfonation at either of the substrate concentrations studied (Table 3). When using 0.1 μM 3-OH-BaP, OH-PCBs with no or one *ortho*-substituted chlorine (B1, B2, B4, C1, C2, C4, and C6) were potent inhibitors of 3-OH-BaP sulfonation, with IC_50_ values between 0.24 and 1.3 μM (Table 3). The OH-PCBs with two *ortho*-substituted chlorine atoms (B3, B5, C3, and C5) were less potent inhibitors, with IC_50_ values of 4.87–7.98 μM (Table 3). When we used 1.0 μM 3-OH-BaP as substrate, there was a 3- to 5-fold reduction in inhibitory potency, and the order of potency remained as it was with 0.1 μM 3-OH-BaP.

### Structure–activity relationships.

**Figure 8:**
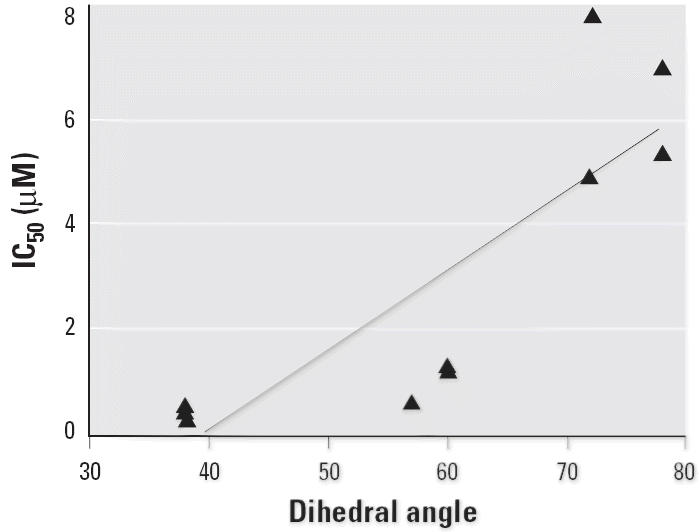
Correlation of IC_50_ values (μM) for type B and type C 4-OH-PCBs with dihedral angles in the presence of SULT1E1. The regression line was significantly different from zero (*p* < 0.001), and the goodness of fit (*r*^2^) was 0.73 for the positive correlation of SULT1E1 IC_50_ values with a dihedral angle.

For HL cytosol, expressed SULT1A1*1, SULT1A1*2, and SULT1E1, we investigated the relationship between inhibitory potency, measured at 0.1 μM 3-OH-BaP, and each of several physicochemical properties of the 4-OH-PCBs. For HL cytosol, SULT1A1*1, and SULT1A1*2, we found no significant correlation between dihedral angle, molecular surface area, molecular surface volume, log *P*, log *D* at pH 7.0, or p*K*_a_. The IC_50_ values with SULT1E1 showed a significant (*p* < 0.001) linear correlation with dihedral angle, as shown in Figure 8. No other significant correlations were found.

### Kinetics of 3-OH-BaP sulfotransferase inhibition by 4′-OH-CB112.

**Figure 9:**
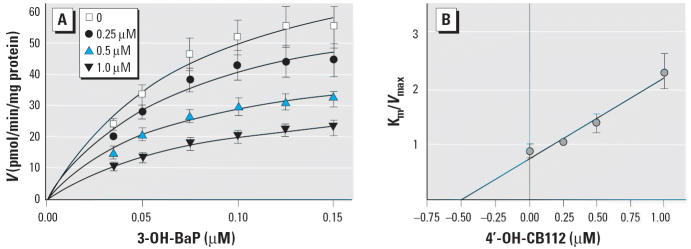
Effect of 4′-OH-CB112 on the kinetics of sulfotransferase with 3-OH-BaP in HL cytosol. (*A*) Saturation curves, with each point representing the mean of data from three livers; the kinetic parameters are summarized in Table 4. (*B*) Plots of apparent *K*_m_/*V*_max_ versus the concentration of 3-OH-BaP for calculation of *K*_i_ value. Data given are the mean ± SD of three experiments.

We investigated the type of inhibition of 3-OH-BaP sulfonation using HL cytosol. Figure 9A shows that 4′-OH-CB112 (B5) reduced sulfotransferase activities at all the tested 3-OH-BaP concentrations in a concentration-dependent manner. The kinetic constants showed a steady reduction in *V*_max_ with increasing concentration of 4′-OH-CB112, but little change in *K*_m_, indicating a noncompetitive type of inhibition (Table 4). Figure 9B shows a plot of *K*_m_/*V*_max_ versus the concentration of 4′-OH-CB112, which indicated a *K*_i_ value for 4′-OH-CB112 of 0.52 ± 0.14 μM.

## Discussion

The major human metabolite of BaP, 3-OH-BaP, was very readily sulfonated in HL cytosol, especially at concentrations < 0.15 μM. We observed substrate inhibition in HL cytosol and with SULT1A1 and SULT1E1, but not with SULT1A3 or SULT1B1. We studied the kinetics of substrate inhibition in liver cytosol and SULT1A1*2 and found that they fit a two-substrate model proposed for the sulfonation of estradiol by SULT1E1. This model suggested that SULT1E1 could bind two molecules of estradiol per molecule of enzyme, one at a preferred site for sulfonation and the other at an allosteric site associated with substrate inhibition (Zhang et al. 1998). Our results suggest a similar scenario for the interaction of 3-OH-BaP with SULT in HL cytosol and SULT1A1. The *K*_m_ values for each of the three tested HL cytosol fractions (48–51 nM), SULT1A1*2 (22 nM), and SULT1A1*1 (18 nM) were low, indicating that 3-OH-BaP has a very high affinity for human SULT1A1. The *K*_i_ values were about 10-fold higher. The 3-OH-BaP was not, however, specific for SULT1A1 but was a substrate for the other human phenol sulfotransferases studied. In particular SULT1E1 showed a high affinity for 3-OH-BaP, with a *K*_m_ of 50 nM. A related compound, 1-hydroxypyrene, also had a very low *K*_m_ with SULT1A1 (8 nM) and SULT1E1 (21 nM) but a higher *K*_m_ with SULT1A3 (0.8 μM) (Ma et al. 2003). When we calculated 3-OH-BaP clearance values (*V*_max_*/K*_m_) for the four partially purified SULT isoforms, the highest value was found for SULT1A1*1 (Table 2). Thus, 3-OH-BaP was a selective but not specific substrate for SULT1A1. Other investigators showed that the SULT1B1 protein content in liver cytosol was about one-fourth that of SULT1A1 (Honma et al. 2002). The present study showed that expressed SULT1B1 had a 40-fold higher *K*_m_ value (2.0 μM) than found in HL cytosol (0.05 μM), so it is not likely to contribute much to HL cytosolic sulfonation of 3-OH-BaP at 0.1 μM substrate concentration (Table 2). Although SULT1A3 had activity with 3-OH-BaP, it is expressed at very low levels in the adult liver (Richard et al. 2001) and is unlikely to contribute much to 3-OH-BaP sulfonation in human liver. Because *K*_m_ values for 3-OH-BaP in HL cytosol were similar to those of purified SULT1A1 and SULT1E1, and others have shown that SULT1A1 is expressed in liver at approximately 14-fold higher concentrations than SULT1E1 (Honma et al. 2002), we conclude that the observed activity with 3-OH-BaP in HL cytosol is catalyzed largely by SULT1A1. Differing structural features for inhibition of SULT1A1 and SULT1E1 by OH-PCBs further support our conclusion that, in HL cytosol, activity with 3-OH-BaP is due primarily to SULT1A1. By chance, the three HL cytosol fractions we used in these studies were from individuals with different *SULT1A1* genotypes. One was SULT1A1*1 homozygous, a second was heterozygous for SULT1A1*1/*2, and the third was SULT1A1*2 homozygous. Kinetic analysis showed little difference among the three cytosol fractions for *V*_1_, which was 121 pmol/min/mg for the homozygous SULT1A1*1 liver and 94 pmol/min/mg protein for the SULT1A1*2 liver (Table 1); however, the small size of our sample precludes a more detailed analysis of genotype effects on 3-OH-BaP sulfonation activities.

In previous studies, we showed that OH-PCBs inhibited 3-OH-BaP sulfonation in catfish intestinal cytosol (van den Hurk et al. 2002) and that a compound structurally related to OH-PCBs, 2,4,4′-trichloro-2′-hydroxydiphenyl ether (triclosan), inhibited sulfonation and glucuronidation of 3-OH-BaP and other substrates in HL cytosol and with SULT1A1, SULT1B1, and SULT1E1 (Wang et al. 2004). Here we demonstrated that a set of 4-OH-PCBs inhibited SULT activity with 3-OH-BaP, the major metabolite of another pollutant chemical, BaP, in HL cytosol as well as with cDNA-expressed SULTs. In HL cytosol, all the 4-OH-PCBs examined inhibited the sulfonation of 3-OH-BaP. Under incubation conditions in which the 3-OH-BaP substrate did not cause substrate inhibition (0.1 μM 3-OH-BaP), compounds with one chlorine atom adjacent to the OH group (B1–B5) were more potent inhibitors of sulfonation than were compounds in type C, with chlorine atoms flank-ing the OH group on each side. We observed very similar results for potency of inhibition and order of inhibitory potency with all three liver cytosol fractions and the two allelic variants of expressed SULT1A1. When incubated with 1.0 μM 3-OH-BaP, a concentration that produced substrate inhibition in liver cytosol and with both SULT1A1 variants, the OH-PCBs were considerably less potent inhibitors in cytosol and even more so with the expressed SULT1A1*1 and SULT1A1*2 enzymes (Table 3 and data not shown). The effect of substrate concentration on the inhibitory potency of the OH-PCBs suggested the possibility that the OH-PCBs competed with the 3-OH-BaP for an inhibitory site of the SULT1A1 protein. Gamage et al. (2003) reported that SULT1A1*2 could accommodate two molecules of the xenobiotic model substrate *p*-nitrophenol in the active site. They proposed that substrate inhibition at high concentrations of *p*-nitrophenol was due to impeded catalysis when both binding sites were occupied. The active site of SULT1A1 appears to be plastic enough to accept a wide range of hydrophobic phenolic compounds (Gamage et al. 2003) and may be able to accommodate two molecules of 3-OH-BaP, leading to substrate inhibition, or one molecule of 3-OH-BaP and one molecule of OH-PCB, resulting in the OH-PCB inhibiting 3-OH-BaP sulfonation. The kinetic studies with 4′-OH-CB112 (B5) showed that the mechanism of inhibition was noncompetitive. This result could fit the scenario for inhibition discussed above but does not suggest direct competition of the OH-PCB for binding to the active site in an orientation that favors sulfonation. Whatever the mechanism of inhibition, the loss in inhibitory potency of OH-PCB when assays were conducted with 1.0 μM 3-OH-BaP suggested that the enzyme favored binding of 3-OH-BaP over binding of OH-PCB, and this was especially true for type C OH-PCBs, which showed a greater loss in potency than did the type B compounds. These findings suggest that OH-PCBs are likely to be poor substrates for sulfonation, but this has not been studied in human liver.

We could not discern any other clear relationship of inhibitory potency with structural features or with physicochemical properties of the OH-PCBs in this relatively small series of compounds, with cytosol or the two expressed SULT1A1 enzymes. The small size of the series of compounds studied and the lack of ready availability of a systematic series of 4-OH-PCBs prevent further analysis of structure–potency relationships at this time.

Of the other expressed enzymes studied, only SULT1E1 exhibited potent inhibition by the 4-hydroxylated PCBs. The structure–inhibitory potency requirement for SULT1E1 was very different from that for HL cytosol, SULT1A1*1, or 1A1*2, where type B compounds were more potent inhibitors than were type C OH-PCBs. With SULT1E1, OH-PCBs with no or one *ortho*-substituted chlorine were more potent as inhibitors of 3-OH-BaP sulfonation than were those with two *ortho*-substituted chlorine atoms. Substituted biphenyls with less than one *ortho* substituent preferentially adopt coplanar conformation of the two phenyl rings, whereas those with two or more *ortho* substituent atoms take on non-coplanar conformations. We found a significant linear correlation between inhibitory potency and calculated solution dihedral angles (Figure 8, Table 3). Similarly, Kester et al. (2000) found that the best OH-PCB inhibitors of estrogen sulfonation (IC_50_ values < 5 nM) did not have chlorine substituents at the 2- or the 6-position. Shevtsov et al. (2003) later showed that 4,4′-di-OH-CB80 (4,4′-di-OH-3,3′,5,5′-tetrachlorobiphenyl) did not bind the SULT1E1 in a planar conformation, but rather with a 30° twist between the phenyl rings. We found that the four OH-PCBs with solution dihedral angles of 38° were more potent inhibitors than were those with larger dihedral angles. Although it is possible that interaction with the protein could alter the conformation of the OH-PCBs, resulting in a different dihedral angle for the enzyme-bound OH-PCB, our results show that lack of *ortho* substituents is associated with higher inhibitory potency for a xenobiotic SULT1E1 substrate, 3-OH-BaP.

SULT1A3 metabolized 3-OH-BaP with a very high *V*_max_, although its preferred substrates are reported to be catecholamines and other monocyclic phenols containing hydrogen bond donors (Dajani et al. 1998). Interestingly, 50 μM OH-PCBs caused little or no inhibition of this enzyme, thereby showing that the inhibitory interaction was enzyme selective. SULT1B1, the thyroid hormone sulfotransferase, catalyzed the sulfonation of 3-OH-BaP; however, OH-PCBs that were potent inhibitors of SULT1A1 were only weak inhibitors of the SULT1B1-catalyzed reaction. In contrast to results with the other enzymes, compound A1 (6′-OH-CB35) was a fairly potent inhibitor of SULT1B1 (Table 3). Previously, *ortho*-, *meta*-, and *para*-hydroxylated PCBs were found to inhibit thyroid hormone sulfonation (Schuur et al. 1998). The *meta*-hydroxylated PCB, 3-OH-2,3′,4,4′,5-pentachlorobiphenyl (3-OH-CB118), was the most potent inhibitor of thyroid hormone sulfonation in male rat liver cytosol, followed by two *para*-hydroxylated PCBs. The *ortho*-hydroxylated PCB had the lowest potency among the four OH-PCBs studied. However, with 3-OH-BaP as substrate, the *ortho*-OH-PCB, 6′-OH-CB35, was a more potent inhibitor than were those with *para*-OH groups, which suggested that the inhibitory interaction with SULT1B1 was substrate dependent.

Because several OH-PCBs have been detected in human blood and are presumably also present in liver and other tissues, it is important to understand their biologic activities. Some OH-PCBs interact with components of thyroid hormone and estrogen hormone systems (Kester et al. 2000; Klasson-Wehler et al. 1993; Schuur et al. 1998; Sinjari and Darnerud 1998). Our finding that OH-PCBs inhibited the sulfonation of 3-OH-BaP in HL suggests another aspect of the toxicology of OH-PCBs. The interaction with phenol sulfotransferase may be of toxicologic importance because sulfonation is a major pathway of xenobiotic biotransformation (Glatt 2002). Sulfonation is particularly important at low concentrations of hydroxylated xenobiotics, such as may be encountered from environmental exposure to pollutants that require CYP-dependent biotransformation to introduce a hydroxyl group before their elimination. Formation of sulfate conjugates of phenolic xenobiotics usually decreases their toxicity, so inhibition of this pathway may lead to prolonged exposure to the parent compound, a shift to an alternative phase II conjugation pathway, glucuronidation, or to further CYP-dependent metabolism. Both 3-OH-BaP and BaP-3-glucuronide bind to hemoglobin (Sugihara and James 2003), a potentially toxic interaction. Further CYP-dependent biotransformation of 3-OH-BaP may lead to more toxic metabolites such as 3-OH-BaP-7,8-dihydrodiol-9,10-oxide (Glatt et al. 1987; Ribeiro et al. 1986). On the other hand, xenobiotics that are activated by sulfonation, such as 2-hydroxyamino-1-methyl-6-phenylimidazo[4,5-*b*]pyridine (Ozawa et al. 1998), may be rendered less toxic in the presence of inhibitors of sulfonation.

Our findings may be placed in the context of the structures of OH-PCBs that have been reported in human blood. All OH-PCB metabolites identified in blood have the hydroxy group in a *para*- or *meta*-position, with chlorine atoms on vicinal carbon atoms (Hovander et al. 2002; Sandau et al. 2000, 2002; Sjodin et al. 2000). The *para*-OH-PCBs found in blood are likely to fall into the type C OH-PCBs examined in this study. Although these were generally less potent as inhibitors of SULT1A1 than the type B OH-PCBs, it is possible that the concentrations of these OH-PCBs may reach inhibitory levels in tissues of highly exposed people or animals. Sjodin et al. (2000) reported total measured OH-PCB concentrations of up to 6 μM in blood lipids, whereas Sandau et al. (2000) reported whole blood concentrations up to 30 nM. Tissue concentrations have not been reported but they may be higher than blood levels. Type B OH-PCBs with the 3-chloro-4-hydroxy substitution pattern do not appear to be persistent in blood; however, of the 209 PCB congeners, 19 have a 3-chloro substitution in one of the phenyl rings, which can be biotransformed to type B OH-PCBs. If type B OH-PCBs are formed in people, their high potency as inhibitors of 3-OH-BaP sulfonation may cause increased toxicity in people who are coexposed to PAH and PCBs.

## Conclusion

We found that several OH-PCBs, especially those with a 3-chloro-4-hydroxy substitution pattern in the phenolic ring, inhibited the sulfonation of 3-OH-BaP in cytosol and with SULT1A1 at submicromolar concentrations. Some OH-PCBs with no or one *ortho* chlorine were potent inhibitors of 3-OH-BaP sulfonation with SULT1E1. SULT1B1- and SULT1A3-catalyzed sulfonation of 3-OH-BaP was less sensitive to inhibition by OH-PCBs. The inhibitory interaction of OH-PCBs with SULT1A1 and SULT1E1 may have consequences for the biotransformation and toxicity of phenolic xenobiotics.

## Figures and Tables

**Table 1:** Kinetic parameters for 3-OH-BaP sulfonation by human livers and SULT1A1*2.

	*K*_m_ (μM)	*K*_i_ (μM)	*V*_1_ (pmol/min/mg protein)	*V*_2_ (pmol/min/mg protein)	*R*^2^
HL 1 cytosol	0.048	0.915	121	2	0.95
HL 2 cytosol	0.051	0.534	93.0	38.50	0.956
HL 3 cytosol	0.048	0.460	94.4	31.0	0.958
Mean ± SD	0.049 ± 0.01	0.636 ± 0.244	102 ± 15.8	28.3 ± 19.3	
SULT1A1*2	0.022	0.160	4,400	290	0.953

Kinetic analysis was performed using a two-substrate model as described in “Materials and Methods.” HL 1 cytosol was homozygous for SULT1A1*1, HL 2 cytosol was heterozygous as SULT1A1*1/*2, and HL 3 cytosol was homozygous for SULT1A1*2.

**Table 2:** Apparent kinetic constants for cDNA-expressed sulfotransferase with 3-OH-BaP as substrate.

SULT	*K*_m_ (μM)	*V*_max_ (nmol/min/mg protein)	*V*_max_/*K*_m_ (mL/min/mg protein)
SULT1A1*1	0.018	6.89	383
SULT1A3	2.90	333.3	115
SULT1B1	2.00	9.70	4.9
SULT1E1	0.05	8.35	167

Partially purified SULT isoforms were used for these studies. *V*_max_ was calculated from the mg/mL of the partially purified preparation and corrected by the percentage of protein estimated to be SULT, from SDS-PAGE: 44.0% for SULT1A1*1, 39.0% for SULT1A3, 80.3% for SULT1B1, and 65.0% for SULT1E1.

**Table 3:** *In vitro* inhibition of 3-OH-BaP sulfotransferase activity by the tested OH-PCBs using HL cytosol and cDNA-expressed sulfotransferases at 0.1 and 1.0 μM substrate concentration.*^a^*

				IC_50_ (μM)							
				0.1 μM 3-OH-BaP				1.0 μM 3-OH-BaP			
Compound no.	Compound	Log *D* at pH 7.0	Dihedral angle (º)	HL cytosol	SULT1A1*1	SULT1A1*2	SULT1E1	HL cytosol	SULT1A1*1	SULT1B1	SULT1E1
A1	6′-OH-CB35	4.7	50	> 100	> 100	> 100	~100	> 100	> 100	4.72	> 100
B1	4′-OH-CB35	4.7	38	0.33 ± 0.02	0.77	0.55	0.24	0.96 ± 0.30	25.2	16.8	1.02
B2	4′-OH-CB36	4.8	38	0.67 ± 0.12	1.31	0.94	0.45	1.05 ± 0.39	28.0	37.0	1.89
B3	4′-OH-CB69	5.1	72	0.91 ± 0.09	1.16	1.31	4.87	1.50 ± 0.32	67.5	> 100	30.8
B4	4′-OH-CB106	5.2	60	0.37 ± 0.04	1.07	1.06	1.18	2.61 ± 0.67	10.3	17.4	6.97
B5	4′-OH-CB112	5.2	78	1.08 ± 0.12	1.17	1.48	5.35	4.22 ± 1.03	42.5	86.5	23.2
C1	4′-OH-CB79	4.5	38	6.71 ± 0.91	6.65	4.57	0.50	58.7 ± 13.9	59.8	39.9	1.32
C2	4-OH-CB36	4.2	38	2.30 ± 0.45	3.09	3.05	0.41	35.9 ± 1.47	> 100	47.5	1.65
C3	4′-OH-CB121	4.7	72	3.95 ± 0.23	8.15	6.52	7.98	44.6 ± 6.42	99.5	> 100	16.7
C4	4′-OH-CB159	4.7	78	1.31 ± 0.14	2.16	1.67	1.30	38.4 ± 15.2	34.1	> 100	3.55
C5	4′-OH-CB165	4.6	78	2.87 ± 0.09	2.58	2.59	6.96	47.3 ± 10.2	54.8	> 100	21.4
C6	4′-OH-CB72	4.5	57	1.72 ± 0.21	2.21	2.03	0.57	3.05 ± 0.41	33.8	> 100	2.28

a Values for HL cytosol are the means ± SDs of three livers, tested in duplicate; results for expressed SULT enzymes are the means of duplicate determinations.

**Table 4:** Apparent kinetic constants for 3-OH-BaP sulfotransferase activity in HL cytosol in the presence and absence of 4′-OH-CB112 (B5).

B5 (μM)	*K*_m_ (μM)	*V*_max_ (pmol/min/mg protein)
0	0.045 ± 0.02 A	69.1 ± 8.0 B
0.25	0.043 ± 0.02 A	56.3 ± 6.0 B
0.5	0.045 ± 0.01 A	41.2 ± 3.3 C
1.0	0.066 ± 0.02 A	33.3 ± 3.2 D

Values for liver cytosol are mean ± SD (*n* = 3), except for studies with 0.25 μM B5, where two livers were used. Different letters indicate values that are significantly different from each other (*p* < 0.05).
